# Expression of aurora kinase A is associated with metastasis-free survival in node-negative breast cancer patients

**DOI:** 10.1186/1471-2407-12-562

**Published:** 2012-11-27

**Authors:** Wulf Siggelkow, Daniel Boehm, Susanne Gebhard, Marco Battista, Isabel Sicking, Antje Lebrecht, Christine Solbach, Birte Hellwig, Jörg Rahnenführer, Heinz Koelbl, Mathias Gehrmann, Rosemarie Marchan, Cristina Cadenas, Jan G Hengstler, Marcus Schmidt

**Affiliations:** 1 Department of Obstetrics and Gynecology, Diakonischen Dienste Hannover GmbH, Diakoniekrankenhaus Henriettenstiftung und Diakoniekrankenhaus Friederikenstift, Hanover, Germany; 2 Department of Obstetrics and Gynecology, Johannes Gutenberg University, Mainz, Germany; 3 Department of Statistics, Dortmund TU, Dortmund, Germany; 4 Bayer GmbH, Leverkusen, Germany; 5 IfADo-Leibniz Research Centre for Working Environment and Human Factors (IfADo), Technical University of Dortmund, Dortmund, Germany; 6 Department of Obstetrics and Gynecology, University of Mainz, Langenbeckstr. 1, Mainz, 55131, Germany

**Keywords:** Aurora kinase, Node-negative breast cancer, Breast cancer, Prognosis, Aurora kinase inhibitors

## Abstract

**Background:**

Inhibitors targeting the cell cycle-regulated aurora kinase A (AURKA) are currently being developed. Here, we examine the prognostic impact of AURKA in node-negative breast cancer patients without adjuvant systemic therapy (n = 766).

**Methods:**

AURKA was analyzed using microarray-based gene-expression data from three independent cohorts of node-negative breast cancer patients. In multivariate Cox analyses, the prognostic impact of age, histological grade, tumor size, estrogen receptor (ER), and HER2 were considered.

**Results:**

Patients with higher AURKA expression had a shorter metastasis-free survival (MFS) in the Mainz (HR 1.93; 95% CI 1.34 – 2.78; P < 0.001), Rotterdam (HR 1.95; 95% CI 1.45– 2.63; P<0.001) and Transbig (HR 1.52; 95% CI 1.14–2.04; P=0.005) cohorts. AURKA was also associated with MFS in the molecular subtype ER+/HER2- carcinomas (HR 2.10; 95% CI 1.70–2.59; P<0.001), but not in ER-/HER2- nor in HER2+ carcinomas. In the multivariate Cox regression adjusted to age, grade and tumor size, AURKA showed independent prognostic significance in the ER+/HER2- subtype (HR 1.73; 95% CI 1.24–2.42; P=0.001). Prognosis of patients in the highest quartile of AURKA expression was particularly poor. In addition, AURKA correlated with the proliferation metagene (R=0.880; P<0.001), showed a positive association with grade (P<0.001), tumor size (P<0.001) and HER2 (P<0.001), and was inversely associated with ER status (P<0.001).

**Conclusions:**

AURKA is associated with worse prognosis in estrogen receptor positive breast carcinomas. Patients with the highest AURKA expression (>75% percentile) have a particularly bad prognosis and may profit from therapy with AURKA inhibitors.

## Background

Aurora kinases A and B are both important for cell cycle progression. They are frequently overexpressed or mutated in human tumor proteins [1,2], and have been implicated in tumor formation and progression [3,4]. Both kinases are highly expressed in several tumor types, including breast, lung, colon, prostate, pancreas, liver, skin, stomach, rectum, esophagus, endometrium, cervix, bladder, ovary, and thyroid cancers compared to the corresponding normal tissues [1,2]. Aurora kinase A (AURKA) is also involved in centrosome function and assembly of the mitotic spindle [5], and has been shown to modulate the activity of tumor suppressors such as p53 [1].

Inhibition of aurora kinase in xenograft models results in tumor regression [6]. Furthermore, inhibitors that target this family of kinases are currently under clinical development. These agents selectively target the enzymatic activity of aurora kinases by occupying the catalytic adenosine triphosphate (ATP)-binding site [7-9].

Several studies have assessed the importance of aurora kinase A and B in breast cancer. In a mouse model, AURKA overexpression was shown to induce breast tumor formation in mammary epithelium [10]. Moreover, polymorphisms in the AURKA gene are associated with increased risk of primary breast cancer [10,11]. This association is synergistic in its effect on the risk of breast cancer in women with prolonged estrogen exposure [12]. AURKA regulates the transition of cells from the G2 to M phase and has been shown to be responsible for the phosphorylation of BRCA1 [13]. Other studies have assessed the expression of AURKA in human breast cancer tissue. For example, Tanaka et al. [14] investigated 33 cases of invasive ductal carcinoma and found AURKA overexpressed in 94% of cases. Miyoshi et al. observed elevated expression in 64% of breast carcinomas using reverse transcription polymerase chain reaction (PCR) in 47 patients [15]. However, a larger study including 112 patients did not find an association between AURKA expression and survival [16]. Furthermore, Nadler et al. observed variable expression of aurora kinase A and B in primary breast tumors [17]. In their study, high levels of AURKA was strongly associated with decreased survival (*P =* 0.0005) and continued to be an independent prognostic marker in the multivariate analysis. High AURKA expression was also associated with high nuclear grade, high HER-2 and progesterone receptor expression. Aurora kinase B expression was not associated with survival [17].

Gene expression profiling has led to a magnitude of different signatures which are related to breast cancer prognosis. In a meta-analysis of publicly available breast cancer gene expression and clinical data, Wiripati and co-workers underscored the important role of proliferation in breast cancer prognosis [18]. Clearly, there are numerous proliferation-associated genes. Martin and co-workers used a novel unsupervised approach to identify a set of genes whose expression predicts prognosis of breast cancer patients [19]. Amongst the most predictive genes for ER positive patients was AURKA, a gene which is a constituent in multiple microarray gene signatures [20-22].

Meanwhile, in a head to head comparison of a large panel of proliferation markers using immunohistochemistry in 3.093 breast carcinomas AURKA outperformed other proliferation markers as an independent predictor of breast cancer-specific survival in ER-positive breast cancer [23]. Finally, a sophisticated analysis of prognostication strategies in breast cancer microarray data sets showed that that the most complex methods were not necessarily better than a univariate model relying on a single gene like AURKA [24]. We could also show that expression of AURKA was associated with survival in node-negative breast cancer in univariate but not in multivariate analysis [25].

In view of the importance of AURKA in malignant progression, together with the current development of aurora kinase inhibitors, we set out to analyze the prognostic significance of AURKA in cohorts of node-negative breast cancer patients who did not receive adjuvant systemic therapy.

## Materials and methods

### Patients

This analysis includes gene array data from node-negative breast cancer patients without adjuvant chemotherapy. The study was approved by the ethical review board of the medical association of Rhineland-Palatinate. The manuscript was prepared in agreement with the reporting recommendations for tumor marker reporting studies [26].

### Gene array data for fresh frozen tissue

Three previously published datasets for untreated node-negative breast cancer patients were used. The large combined group of 766 patients included the Mainz cohort with 200 patients (Table 1) [27], the Rotterdam cohort with 286 patients (Table 2) [28], and the TRANSBIG cohort with 280 patients (Table 3) [29,30]. These cohorts comprise available microarray datasets for medically untreated node-negative breast cancer which have used metastasis-free survival (MFS) as an end point.

**Table 1:** Clinicopathological characteristics of node negative breast cancer patients (fresh frozen tissue) from the Mainz cohort (n=200)

**Characteristics**	**n**	%
Age at diagnosis		
<50	49	24.5
≥50	151	75.5
pT stage		
≤2cm	106	53.0
>2cm	88	44.0
not documented	6	3.0
Histological grade		
G I	42	21.0
G II	109	54.5
G III	49	24.5
Estrogen receptor status^1^		
RNA expression low	31	15.5
RNA expression high	169	84.5
Progesterone receptor status^1^		
RNA expression low		
RNA expression high	86	43.0
	114	57.0
Hormone receptor status^2^		
RNA expression low		
RNA expression high	31	15.5
	169	84.5
HER2 status^1^		
RNA expression low	181	90.5
RNA expression high	19	9.5
Metastasis		
Yes	47	23.5
No	153	76.5

^1^Estrogen, progesterone and HER2 status were derived from RNA levels as described in Schmidt et al., 2010 [34]. ^2^The hormone receptor status is defined as positive when one of either the estrogen or the progesterone receptor status is positive.

**Table 2:** Clinicopathological characteristics of node negative breast cancer patients (fresh frozen tissue) from the Rotterdam cohort (n=286)

**Characteristics**	**n**	%
Estrogen receptor		
RNA expression low	78	27.3
RNA expression high	208	72.7
Progesterone receptor		
RNA expression low	158	55.2
RNA expression high	128	44.8
Hormone receptor status^1^		
RNA expression low		
RNA expression high	76	26.6
	210	73.4
HER2 status		
RNA expression low	236	82.5
RNA expression high	50	17.5
Metastasis		
Yes	179	62.6
No	107	37.4

Estrogen receptor, progesterone receptor and HER-2 status were derived from the gene array data. Cut-points were 8.2 for the estrogen receptor, 11.2 for HER-2, and 4.5 for the progesterone receptor. Log2 transformed gene array data have been used.

^1^The hormone receptor status is positive when either the estrogen or progesterone receptor RNA expression is high.

**Table 3:** Clinicopathological characteristics of node negative breast cancer patients (fresh frozen tissue) from the Transbig cohort (n=280)

**Characteristics**	**n**	%
Age at diagnosis		
<50	158	56.4
≥50	122	43.5
pT stage		
≤2cm	107	38.2
>2cm	173	61.8
Histological grade		
G I+II	165100	58.9
G III	15	35.7
not documented		5.4
Estrogen receptor		
RNA expression low	79	28.2
RNA expression high	201	71.8
Progesterone receptor		
RNA expression low	156	55.7
RNA expression high	124	43.3
Hormone receptor status^1^		
Negative		
Positive	78	27.9
	202	72.1
HER2 status		
RNA expression low	245	87.5
RNA expression high	35	12.5
Metastasis		
Yes	72	74.3
No	208	25.7

Estrogen receptor, progesterone receptor and HER-2 status were derived from the gene array data. Cutpoints were 8.2 for the estrogen receptor, 10.2 for HER-2, and 4.5 for the progesterone receptor. Log2 transformed gene array data have been used.

^1^The hormone receptor status is positive when either the estrogen or progesterone receptor RNA expression is high.

### Gene expression profiling and data processing

For the Mainz, Rotterdam, and TRANSBIG cohorts, the Affymetrix, Inc. (Santa Clara, California) Human Genome U133A Array set and GeneChip System^TM^ were used to quantify the relative transcript abundance in the breast cancer tissues, as previously described [27], and the robust multiarray average (RMA) algorithm was used for normalization. To analyze AURKA expression from the gene array data, probe set ID 204092_s_at was used in all cohorts*.*

### Statistical analysis

Survival rates were calculated using the Kaplan–Meier method. Metastasis-free survival was computed from the date of diagnosis to the date of distant metastasis. Survival functions were compared with the Log-rank test. Multivariate Cox survival analyses were performed with inclusion. Categorization was performed as follows: aurora kinase mRNA: < median, ≥ median; age: < 50 years, ≥ 50 years; HER-2 status, ER status, PR status: negative, positive; histological grade: GI and GII, GIII; pT stage: pT1 (≤ 2 cm), pT2 and pT3 (> 2 cm). Hormone receptor status was dichotomized on the basis of the corresponding gene expression values. All p-values are two-sided. As no correction for multiple testing was performed they are descriptive measures. All analyses were performed using R2.12.1.

## Results

**Figure 1:**
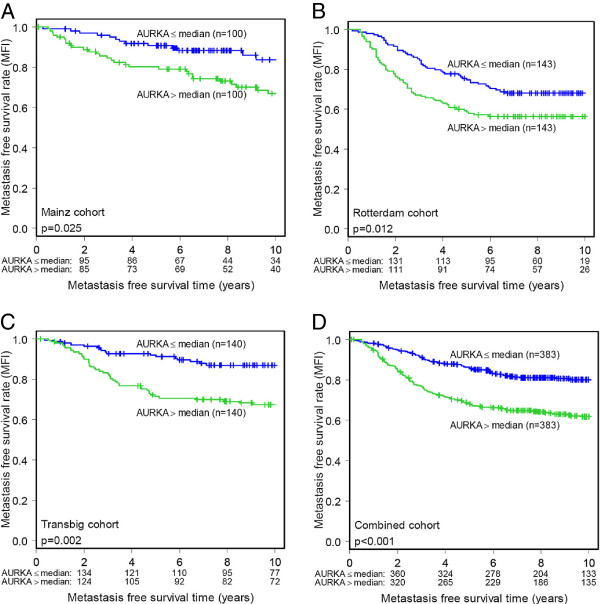
**Metastasis-free survival (MFS) in relation to AURKA expression in the individual subcohorts (Mainz, Rotterdam and Transbig) and in the combined cohort. A**. Mainz cohort (n=200), **B**. Rotterdam cohort (n=286), **C**. Transbig cohort (n=280), and **D**. combined cohort (n=766).

**Figure 2:**
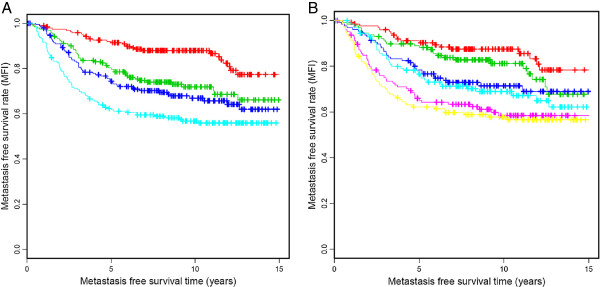
**Relationship between AURKA levels in breast carcinomas and metastasis-free survival (MFS). A**. Patients were subdivided into four percentiles with increasing AURKA expression and analyzed by Kaplan-Meier plots. Red, green, dark blue, light blue represent the 1^st^, 2^nd^, 3^rd^ and 4^th^ quartiles of AURKA expression, respectively. **B**. Similarly, six groups of equal case numbers were analyzed. The colors red, green, dark blue, light blue and yellow show groups of patients with increasing AURKA expression.

To study the prognostic impact of AURKA, we used three publicly accessible Affymetrix gene array data sets, more specifically only node-negative breast cancer patients who did not receive chemotherapy: the Mainz, Rotterdam, and Transbig cohorts (Tables 1, 2, and 3) [27-30]. Expression of AURKA was detectable in all carcinomas and showed a unimodal distribution. AURKA was associated with metastasis-free interval (MFI) in the combined cohort as well as in all three subcohorts using the univariate Cox analysis (Table 4). Similarly, Kaplan-Meier analysis showed a strong association between high AURKA expression and shorter MFI (Figure 1). Next, we studied whether patients with the highest expression levels of AURKA suffer from a particularly high risk of metastasis. For this purpose, we subdivided the 766 patients into four (Figure 2A) and six (Figure 2B) equal groups with increasing levels of AURKA. This analysis illustrates that patients with AURKA levels between the 25 and 50% percentile suffer from shorter metastasis-free survival than patients with expression below the 25% percentile. The 25% of carcinomas with the highest expression (>75% percentile) have the worse prognosis (Figure 2A). Additional subdivision into six groups of equal size did not allow a further differentiation (Figure 2B).

**Table 4:** AURKA is associated with metastasis-free survival (MFS) in three independent cohorts of systemically untreated node negative breast cancer (combined Mainz, Rotterdam and Transbig cohorts, n=766)

		**Mainz cohort(n=200)**	**Rotterdam cohort(n=286)**	**Transbig cohort(n=280)**	**Combined cohorts(n=766)**
A. Univariate Cox analysis					
AURKA	P-value	<0.001	<0.001	0.005	<0.001
	HR	1.927	1.952	1.520	1.669
	95%-CI	1.335-2.782	1.448-2.632	1.135-2.036	1.402-1.986
B. Multivariate Cox analysis of MFS adjusted to established clinical factors (combined Mainz and Transbig cohorts, n=465)					
		**p**	**HR**	**95%CI**	
Age (<50 *vs.* ≥50 years)		0.392	1.180	0.808-1.726	
pT stage (≤2cm *vs.* >2cm)		0.005	1.812	1.192-2.754	
Histological grade (Grade 1 and 2 *vs.* grade 3)		0.087	1.529	0.940-2.487	
ER status (negative *vs.* positive)		0.413	1.214	0.763-1.931	
HER2 status (negative *vs.* positive)		0.415	1.248	0.732-2.128	
AURKA (continuous variable)		0.046	1.350	1.005-1.812	

HR: hazards ratio, 95%-CI: 95% confidence interval. AURKA was analyzed as a continuous variable.

Breast cancer is not a homogeneous disease, making it necessary to differentiate among the different molecular subtypes. A frequently applied system was introduced by Desmedt et al., differentiating between ER+/HER2-, ER-/HER2- and HER2+ carcinomas [31]. Interestingly, only the ER+/HER2- molecular subtype showed an association between AURKA and MFI, a result relevant for the total cohort (Table 4), as well as for each of the three subcohorts. In contrast, AURKA was not significantly associated with MFI in the ER-/HER2- and in the HER2+ carcinomas, respectively. Using multivariate Cox analysis adjusted to age, pTstage and histological grade, AURKA was also significantly associated with MFI in the ER+/HER2- (Table 5) but not in the ER-/HER2- (Table 6) carcinomas. The association in the HER2+ subgroup (Table 6) should be interpreted with caution because of the small case number.

**Table 5:** **Cox analysis of metastasis-free survival (MFS) in the molecular subtypes (ER+/HER; ER-/HER2-; HER2+) according to Desmedt and co-workers [**[31]**]**

	**Mainz cohort(n=158)**	**Rotterdam cohort(n=178)**	**Transbig cohort(n=186)**	**Combined cohorts(n=522)**	
A. Univariate analysis					
ER+/HER2-					
AURKA	P-value	0.011	<0.001	<0.001	<0.001
	HR	1.786	2.916	2.174	2.100
	95%-CI	1.144-2.787	2.022-4.206	1.491-3.171	1.700-2.594
	**Mainz cohort(n=23)**	**Rotterdam cohort(n=58)**	**Transbig cohort(n=59)**	**Combined cohorts(n=140)**	
ER-/HER2-					
AURKA	P-value	0.497	0.808	0.924	0.993
	HR	1.534	1.103	0.967	1.002
	95%-CI	0.446-5.282	0.498-2.443	0.483-1.934	0.637-1.577
	**Mainz cohort(n=19)**	**Rotterdam cohort (n=50=)**	**Transbig cohort (n=35)**	**Combined cohorts (n=104)**	
HER2+					
AURKA	P-value	0.298	0.840	0.100	0.402
	HR	2.303	1.088	0.439	0.785
	95%-CI	0.478-11.091	0.481-2.461	0.165-1.171	0.446-1.382

AURKA is associated with MFS in the estrogen receptor positive but not in the estrogen receptor negative subtypes. A. Univarate analysis.

**Table 6:** **Cox analysis of metastasis-free survival (MFS) in the molecular subtypes (ER+/HER; ER-/HER2-; HER2+) according to Desmedt and co-workers [**[31]**]**

	**p**	**HR**	**95%CI**
B. Multivariate analysis			
ER+/HER2- (n=332)			
Age (<50 *vs.* ≥50 years)	0.920	1.025	0.634-1.656
pT stage (≤2cm *vs.* >2cm)	0.004	2.143	1.271-3.613
Histological grade (Grade 1 and 2 *vs.* grade 3)	0.456	1.255	0.691-2.283
AURKA (continuous variable)	0.001	1.734	1.242-2.419
	**p**	**HR**	**95%CI**
ER-/HER2- (n=80)			
Age (<50 *vs.* ≥50 years)	0.644	0.825	0.365-1.865
pT stage (≤2cm *vs.* >2cm)	0.466	1.459	0.528-4.028
Histological grade (Grade 1 and 2 *vs.* grade 3)	0.590	0.768	0.294-2.005
AURKA (continuous variable)	0.858	0.943	0.497-1.791
	**p**	**HR**	**95%CI**
HER2+ (n=53)			
Age (<50 *vs.* ≥50 years)	0.018	5.072	1.327-19.383
pT stage (≤2cm *vs.* >2cm)	0.485	1.510	0.475-4.802
Histological grade (Grade 1 and 2 *vs.* grade 3)	0.001	15.527	3.223-74.793
AURKA (continuous variable)	0.001	0.146	0.045-0.466

AURKA is associated with MFS in the estrogen receptor positive but not in the estrogen receptor negative subtypes. B. Multivariate Cox regression.

**Figure 3:**
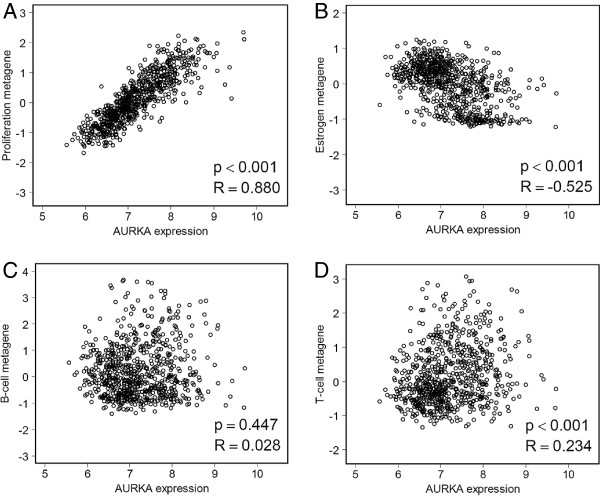
**Correlation of AURKA expression with biological motifs expressed by metagenes [**[27]**]:A. Proliferation, B. Estrogen receptor,C. -cell and D. T-cell metagenes.**

**Figure 4:**
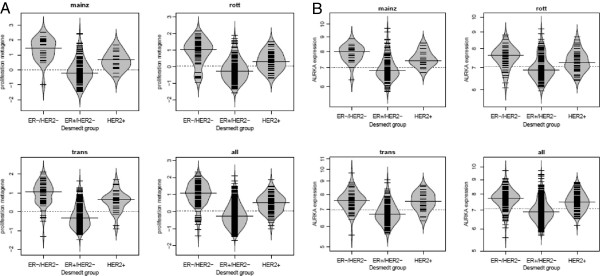
**Beanplots showing expression levels of AURKA (A) and the proliferation metagene (B) in the three different molecular subtypes of breast cancer in each individual subcohort (Mainz, Rotterdam and Transbig) and in the combined cohort (all).** The small lines represent the data points. The median is represented by a longer line.

Recently, Schmidt and co-workers have identified metagenes that represent biological motifs - proliferation, estrogen receptor and immune system - in breast cancer [27]. AURKA shows a strong correlation with the proliferation metagene (Figure 3A). A weaker inverse correlation was obtained with estrogen receptor-associated genes (Figure 3B). No or only extremely weak correlations were obtained with the B- and T-cell metagenes, respectively (Figure 3C, Figure 3D). In addition, AURKA RNA levels correlated with histological grade (P<0.001), tumor size (P<0.001) and HER2 (P<0.001). Considering the molecular subtypes, AURKA showed higher mRNA levels in ER-/HER2- and HER2+ tumors, whereas expression was lower in ER+/HER2- carcinomas (Figure 4A). A similar pattern was observed for the proliferation metagene (Figure 4B). Similarly as observed for AURKA, also the proliferation metagene was associated with MFI in ER+/HER2- but not in ER-/HER2- nor in HER2+ carcinomas (Additional file 1: Table S1). In conclusion, the correlation of AURKA with metagenes and clinical factors reflects the characteristic pattern of a proliferation-associated gene.

**Figure 5:**
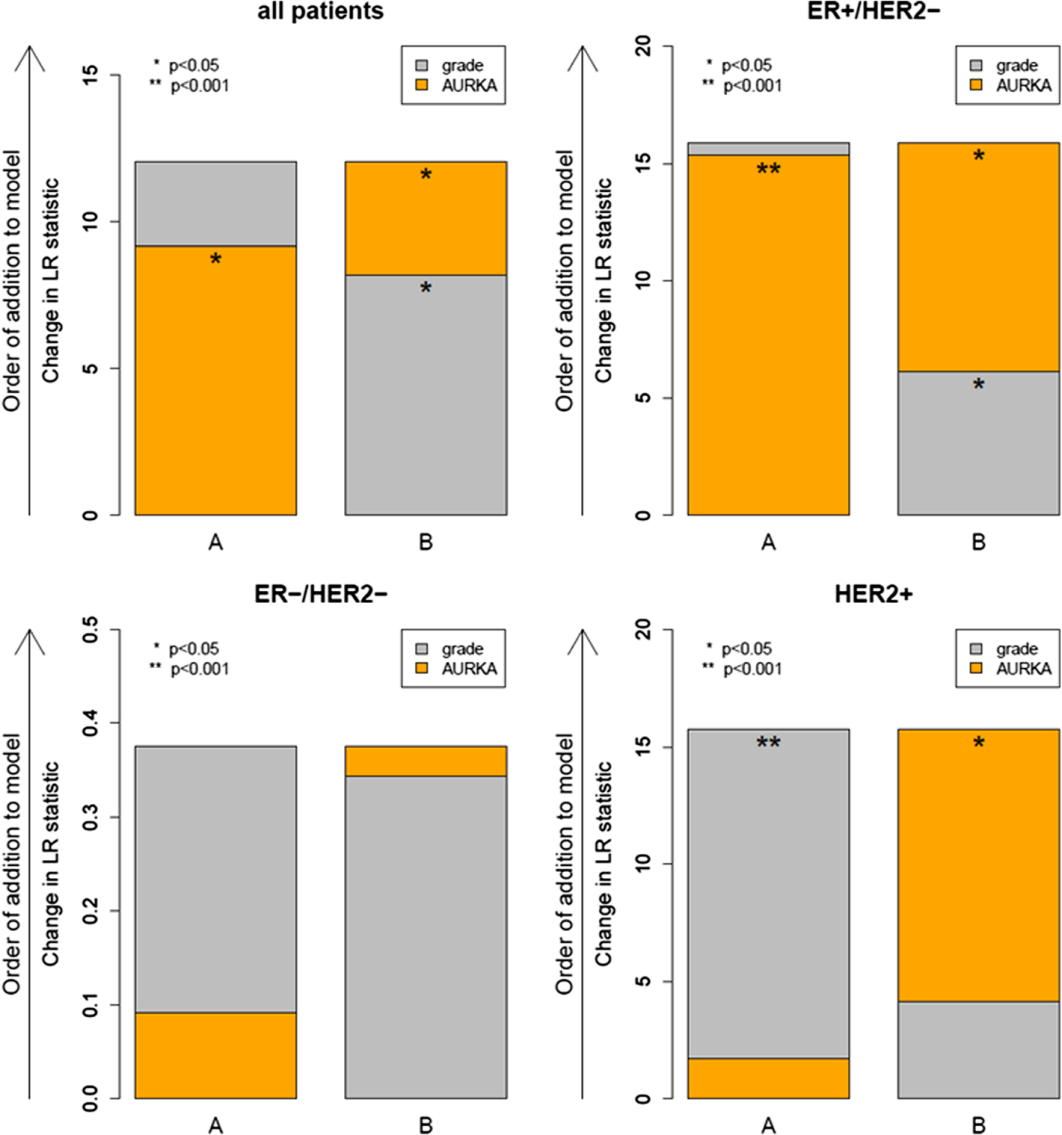
**Metastasis free survival likeliehood statistics as described by Prat et al., [**[32]**].** To compare the amount of independent prognostic information provided by grading (A) and AURKA (B) we estimated the likelihood ratio statistic in a model that already included AURKA (A) or grading (B). The model shows that AURKA provides significant additional information over grading in the cohort of all patients, as well as in the ER+/HER2- and in the HER2+ subgroups (B). Vice versa, grading provides additional information over AURKA only in the subcohort ofHER2+ patients (A).

Given the high correlation of AURKA and histological grade and the association of grading with prognosis we analyzed whether there is a real benefit of considering AURKA expression. For this purpose we performed an analysis similarly as Prat and co-workers [32]. To compare the amount of independent prognostic information provided by AURKA we estimated the likelihood ratio statistic in a model that already included grading (Figure 5). The model showed that AURKA provided significant additional information over grading in the cohort of all patients, as well as in the ER+/HER2- and in the HER2+ subgroups. In previous publications Ep-CAM was described as strong prognostic factor in breast cancer [33,34]. The likelihood ratio statistic shows that AURKA also adds independent prognostic information over Ep-CAM in the cohort of all patients as well as in the ER+/HER2- subgroup Additional file 2: Figure S1.

In the present study the Affymetrix probe set 204092_s_at was used as a measure of AURKA expression. However, similar results were obtained also with the probe set 208079_s_at, which highly correlates with 204092_s_at (R=0.920; P<0.001) (Additional file 1: Figure S1 and Additional file 1: Table S2). A third probe set (208080_at) did not correlate with the other probe sets and should therefore be treated with caution.

## Discussion

Currently, inhibitors of aurora kinases are under preclinical and clinical development [6,35,36]. However, the available data on whether high AURKA expression is associated with worse prognosis in breast cancer remain controversial. Nadler et al. [17] reported an association with survival; however, another study with 112 patients was unable to confirm this result [16]. The discrepancy might be explained by the relatively small case numbers. Therefore, we used a well-established cohort of 766 node-negative breast cancer patients [27] to clarify whether AURKA is prognostic. This cohort did not receive chemotherapy, and therefore provides ideal conditions to study the natural course of the disease. In our initial analysis, AURKA was not independently associated with survival in the whole cohort of patients [25].

The present study demonstrates that high AURKA expression is associated with worse prognosis in univariate analysis. AURKA was not only significant in the total (combined) cohort, but also in each of the three individual subcohorts (Mainz, Rotterdam, Transbig) that were recruited at different centers. Besides showing an association in the univariate Cox model, AURKA was also significant in the multivariate regression adjusted to conventional clinical parameters. However, it should be considered that AURKA performed differently in the three molecular subtypes of breast cancer. Whereas a significant association was obtained in the ER+/HER2- carcinomas, no association with prognosis was seen in the ER-/HER2- and in the HER2+ carcinomas. The strong prognostic impact of AURKA in ER+/HER2- carcinomas is in agreement with the recent observation made by Haibe-Kains and co-workers [37]. These authors used AURKA in addition to ER and HER2 to robustly define breast cancer subtypes. Expression of AURKA distinguished ER+/HER2- low-risk luminal A like carcinomas from ER+/HER2- high-risk luminal B like carcinomas. In addition to this finding, the different result in estrogen receptor positive and negative patients may have important clinical implications. It is tempting to speculate that aurora kinase A inhibitors may be less efficient in estrogen receptor negative carcinomas where AURKA is not associated with prognosis.

Our findings concerning the different performance of AURKA in the different molecular breast cancer subtypes may explain the contradictory results on the prognostic role of AURKA in the studies of Royce et al. [16] and Nadler et al. [17]. Royce and co-workers did not observe an association of AURKA with survival. However, this study included a relatively high fraction of ER- patients (33% ER and/or PR positive, 32.1% ER and PR negative, 34.8% unknown). In contrast, in our study 79%, 62.2% and 66.4% of the patients were ER+ in the Mainz, Rotterdam and Transbig cohorts, respectively. The study group of Nadler also included a relatively high fraction of hormone receptor positive (52% ER+ and 46% PR+) patients. Therefore, the different numbers of hormone receptor-positive patients in the individual groups may explain the discrepancy.

To illustrate the biological function of AURKA, we analyzed its correlation with metagenes of biological motifs [27]. AURKA strongly correlated with the proliferation metagene. Conversely, no relevant correlations were obtained with the immune (B- and T-cell) metagenes. Therefore, AURKA seems to reflect the degree of proliferation of carcinomas which is in agreement with its biological function in mitosis [2,5].

It might appear controversial that AURKA is not significantly associated with worse prognosis in ER-/HER2- and HER2+ tumors although they express even higher levels of AURKA and the proliferation metagene. However, previous studies have already demonstrated that other biological motifs are relevant for the prognosis of ER- and HER2+ carcinomas, particularly an immune cell signature [27] which is best represented by IGKC as a biomarker [38].

## Conclusion

We have shown that AURKA is prognostic in breast cancer patients who did not receive chemotherapy. The prognostic impact of AURKA is most significant in the ER+/HER2- molecular subgroup. The present study has two potential implications for clinical studies with AURKA inhibitors: (i) ER+ patients seem more suitable. (ii) Carcinomas with the highest levels (>75% percentile) of AURKA showed a particularly poor prognosis. Therefore, monitoring AURKA expression will be especially beneficial for patients with high AURKA levels who may profit from chemotherapy with AURKA inhibitors.

## Competing interests

The authors declare that they have no competing interests.

## Authors’ contributions

MS, HK, JGH, MG conceived and designed the experiments. WS, MS, CC, SG, JGH, MG performed the experiments. WS, MS, BH, JGH, CC, JR, SG, DB, CS, AL, MJB, IS, CC, RM, analyzed the data. WS, MS, JGH, CC wrote the paper. All authors read and approved the final manuscript.

## Authors’ information

Jan G Hengstler and Marcus Schmidt shared senior authorship.

## Pre-publication history

The pre-publication history for this paper can be accessed here:

http://www.biomedcentral.com/1471-2407/12/562/prepub

## Supplementary Material

Additional file 1 **Table S1.** Cox analysis of metastasis free survival (MFS) in the single cohorts, in the combined cohort and in the molecular subtypes (ER+/HER2-, ER-/HER2-, HER2+) according to Desmedt (2008). The proliferation metagene is associated with MFI in the estrogen receptor positive but not in the estrogen receptor negative subtypes. **Figure S1**: Scatter plots showing correlation of AURKA probe sets. Whereas *208079_s_at* and *204092_s_at* highly correlate with each other the probe set *208080_at* shows poor correlation with the other two. **Table S2**: Similarly as the probe set described in the main manuscript (204092_s_at) the AURKA probe set 208079_s_at is associated with metastasis-free survival (MFS) in the three independent cohorts of systemically untreated node negative breast cancer (combined Mainz, Rotterdam and Transbig cohorts, n=766). HR: hazards ratio, 95%-CI: 95% confidence interval. AURKA was analyzed as a continuous variable. **Table S3**: Cox analysis of metastasis-free survival (MFS) in the molecular subtypes (ER+/HER; ER-/HER2-; HER2+) according to Desmedt (2008). The AURKA probe set *208079_s_at* is associated with MFI in the estrogen receptor positive but not in the estrogen receptor negative subtypes, as described for 204092_s_at in the main manuscript. A. Univariate analysis, B. Multivariate Cox regression (DOC 162 kb) Click here for file

Additional file 2: **Figure S1.** Metastasis free survival likeliehood statistics as described by Prat et al., (2012). To compare the amount of independent prognostic information provided by Ep-CAM (**A**) and AURKA (**B**) we estimated the likelihood ratio statistic in a model that already included AURKA (A) or Ep-CAM (**B**). The model shows that AURKA provides significant additional information over grading in the cohort of all patients, as well as in the ER+/HER2- subgroups (B). Vice versa, Ep-CAM provides additional information over AURKA only in the cohort of all patients. (PPT 182 kb) Click here for file
