# Supplementary material for: Expression of aurora kinase A is associated with metastasis-free survival in node-negative breast cancer patients
Source: BMC Cancer. 2012 Nov 27;12:562. doi: 10.1186/1471-2407-12-562 (PMC3530429; doi:10.1186/1471-2407-12-562)
Supplement: Additional file 1 — Table S1. Cox analysis of metastasis free survival (MFS) in the single cohorts, in the combined cohort and in the molecular subtypes (ER+/HER2-, ER-/HER2-, HER2+) according to Desmedt (2008). The proliferation metagene is associated with MFI in the estrogen receptor positive but not in the estrogen receptor negative subtypes. Figure S1: Scatter plots showing correlation of AURKA probe sets. Whereas 208079_s_at and 204092_s_at highly correlate with each other the probe set 208080_at shows poor correlation with the other two. Table S2: Similarly as the probe set described in the main manuscript (204092_s_at) the AURKA probe set 208079_s_at is associated with metastasis-free survival (MFS) in the three independent cohorts of systemically untreated node negative breast cancer (combined Mainz, Rotterdam and Transbig cohorts, n=766). HR: hazards ratio, 95%-CI: 95% confidence interval. AURKA was analyzed as a continuous variable. Table S3: Cox analysis of metastasis-free survival (MFS) in the molecular subtypes (ER+/HER; ER-/HER2-; HER2+) according to Desmedt (2008). The AURKA probe set 208079_s_at is associated with MFI in the estrogen receptor positive but not in the estrogen receptor negative subtypes, as described for 204092_s_at in the main manuscript. A. Univariate analysis, B. Multivariate Cox regression (DOC 162 kb) [file 1471-2407-12-562-S1.doc]

**Supplementary information**

Table 1: Cox analysis of metastasis free survival (MFS) in the single cohorts, in the combined cohort and in the molecular subtypes (ER+/HER2-, ER-/HER2-, HER2+) according to Desmedt (2008). The proliferation metagene is associated with MFI in the estrogen receptor positive but not in the estrogen receptor negative subtypes.

**A.Univariate Cox analysis in the combined cohort**

|  | | **Mainz cohort**  **(n=200)** | **Rotterdam cohort (n=286)** | **Transbig cohort (n=280)** | **Combined cohorts (n=766)** |
| --- | --- | --- | --- | --- | --- |
| **Proliferation metagene** | **P-value**  **HR**  **95%-CI** | **<0.001**  **1.966**  **1.440-2.683** | **0.001**  **1.505**  **1.195-1.895** | **0.012**  **1.425**  **1.081-1.880** | **<0.001**  **1.586**  **1.359-1.851** |

**B. Univariate Cox analysis in ER+/HER2-**

|  | | **Mainz cohort**  **(n=158)** | **Rotterdam cohort (n=178)** | **Transbig cohort (n=186)** | **Combined cohorts (n=522)** |
| --- | --- | --- | --- | --- | --- |
| **Proliferation metagene** | **P-value**  **HR**  **95%-CI** | **0.001**  **1.995**  **1.307-3.047** | **<0.001**  **2.367**  **1.675-3.344** | **<0.001**  **2.686**  **1.753-4.117** | **<0.001**  **2.344**  **1.868-2.940** |

**C. Univariate Cox analysis in ER-/HER2-**

|  | | **Mainz cohort**  **(n=23)** | **Rotterdam cohort (n=58)** | **Transbig cohort (n=59)** | **Combined cohorts (n=140)** |
| --- | --- | --- | --- | --- | --- |
| **Proliferation metagene** | **P-value**  **HR**  **95%-CI** | **0.625**  **1.267**  **0.490-3.275** | **0.981**  **1.007**  **0.581-1.744** | **0.185**  **0.668**  **0.369-1.212** | **0.513**  **0.882**  **0.602-1.290** |

**D. Univariate Cox analysis in HER2+**

|  | | **Mainz cohort**  **(n=19)** | **Rotterdam cohort (n=50)** | **Transbig cohort (n=35)** | **Combined cohorts (n=104)** |
| --- | --- | --- | --- | --- | --- |
| **Proliferation metagene** | **P-value**  **HR**  **95%-CI** | **0.006**  **12.581**  **2.046-77.345** | **0.522**  **1.299**  **0.583-2.891** | **0.065**  **0.420**  **0.167-1.057** | **0.982**  **0.993**  **0.555-1.778** |

**Supplemental Fig. 1:** Scatter plots showing correlation of AURKA probe sets. Whereas *208079_s_at* and *204092_s_at* highly correlate with each other the probe set *208080_at* shows poor correlation with the other two.

**Table 2:** Similarly as the probe set described in the main manuscript (204092_s_at) the AURKA probe set 208079_s_at is associated with metastasis-free survival (MFS) in the three independent cohorts of systemically untreated node negative breast cancer (combined Mainz, Rotterdam and Transbig cohorts, n=766). HR: hazards ratio, 95%-CI: 95% confidence interval. AURKA was analyzed as a continuous variable.

**A. Univariate Cox analysis**

|  | | **Mainz cohort**  **(n=200)** | **Rotterdam cohort (n=286)** | **Transbig cohort (n=280)** | **Combined cohorts (n=766)** |
| --- | --- | --- | --- | --- | --- |
| **AURKA** | **P-value**  **HR**  **95%-CI** | **<0.001**  **1.817**  **1.374-2.403** | **<0.001**  **1.541**  **1.263-1.880** | **0.011**  **1.325**  **1.068-1.644** | **<0.001**  **1.532**  **1.345-1.744** |

**B. Multivariate Cox analysis of MFI adjusted to established clinical factors (combined Mainz and Transbig cohorts, n=465)**

|  | **p** | **HR** | **95% CI** |
| --- | --- | --- | --- |
| **Age**  **(<50 *vs.* ≥50 years)** | **0.203** | **1.282** | **0.875-1.878** |
| **pT stage**  **(≤2cm *vs.* >2cm)** | **0.007** | **1.776** | **1.167-2.703** |
| **Histological grade**  **(Grade 1 and 2 *vs.* grade 3)** | **0.102** | **1.507** | **0.922-2.464** |
| **ER status**  **(negative *vs.* positive)** | **0.394** | **1.224** | **0.769-1.947** |
| **HER2 status**  **(negative *vs.* positive)** | **0.452** | **1.228** | **0.719-2.097** |
| **AURKA**  **(continuous variable)** | **0.039** | **1.258** | **1.011-1.565** |

**Table 3:** Cox analysis of metastasis-free survival (MFS) in the molecular subtypes (ER+/HER; ER-/HER2-; HER2+) according to Desmedt (2008). The AURKA probe set *208079_s_at* is associated with MFI in the estrogen receptor positive but not in the estrogen receptor negative subtypes, as described for 204092_s_at in the main manuscript. A. Univariate analysis, B. Multivariate Cox regression

**A. Univariate Cox analysis in the Desmedt subtypes**

**ER+/HER2-**

|  | | **Mainz cohort**  **(n=158)** | **Rotterdam cohort (n=178)** | **Transbig cohort (n=186)** | **Combined cohorts (n=522)** |
| --- | --- | --- | --- | --- | --- |
| **AURKA** | **P-value**  **HR**  **95%-CI** | **0.001**  **1.774**  **1.270-2.477** | **<0.001**  **2.112**  **1.633-2.732** | **<0.001**  **1.642**  **1.251-2.156** | **<0.001**  **1.863**  **1.585-2.189** |

ER-/HER2-

|  | | **Mainz cohort**  **(n=23)** | **Rotterdam cohort (n=58)** | **Transbig cohort (n=59)** | **Combined cohorts (n=140)** |
| --- | --- | --- | --- | --- | --- |
| **AURKA** | **P-value**  **HR**  **95%-CI** | **0.363**  **1.540**  **0.607-3.911** | **0.927**  **1.026**  **0.595-1.767** | **0.811**  **0.936**  **0.544-1.610** | **0.765**  **1.056**  **0.738-1.511** |

**HER2+**

|  | | **Mainz cohort**  **(n=19)** | **Rotterdam cohort (n=50)** | **Transbig cohort (n=35)** | **Combined cohorts (n=104)** |
| --- | --- | --- | --- | --- | --- |
| **AURKA** | **P-value**  **HR**  **95%-CI** | **0.247**  **1.765**  **0.675-4.619** | **0.831**  **1.060**  **0.619-1.816** | **0.071**  **0.498**  **0.234-1.061** | **0.493**  **0.866**  **0.574-1.307** |

**B. Multivariate Cox analysis in the Desmedt subtypes**

**ER+/HER2- (n=332)**

|  | **p** | **HR** | **95% CI** |
| --- | --- | --- | --- |
| **Age**  **(<50 *vs.* ≥50 years)** | **0.415** | **1.221** | **0.756-1.971** |
| **pT stage**  **(≤2cm *vs.* >2cm)** | **0.005** | **2.128** | **1.261-3.591** |
| **Histological grade**  **(Grade 1 and 2 *vs.* grade 3)** | **0.548** | **1.203** | **0.658-2.200** |
| **AURKA**  **(continuous variable)** | **<0.001** | **1.549** | **1.213-1.977** |

**ER-/HER2- (n=80)**

|  | **p** | **HR** | **95% CI** |
| --- | --- | --- | --- |
| **Age**  **(<50 *vs.* ≥50 years)** | **0.605** | **0.803** | **0.350-1.842** |
| **pT stage**  **(≤2cm *vs.* >2cm)** | **0.437** | **1.505** | **0.536-4.224** |
| **Histological grade**  **(Grade 1 and 2 *vs.* grade 3)** | **0.632** | **0.790** | **0.300-2.077** |
| **AURKA**  **(continuous variable)** | **0.707** | **0.907** | **0.544-1.512** |

**HER2+ (n=53)**

|  | **p** | **HR** | **95% CI** |
| --- | --- | --- | --- |
| **Age**  **(<50 *vs.* ≥50 years)** | **0.197** | **2.170** | **0.669-7.039** |
| **pT stage**  **(≤2cm *vs.* >2cm)** | **0.208** | **2.169** | **0.649-7.250** |
| **Histological grade**  **(Grade 1 and 2 *vs.* grade 3)** | **0.001** | **10.869** | **2.631-44.894** |
| **AURKA**  **(continuous variable)** | **0.002** | **0.286** | **0.127-0.642** |
