# Supplementary material for: Expression of aurora kinase A is associated with metastasis-free survival in node-negative breast cancer patients
Source: BMC Cancer. 2012 Nov 27;12:562. doi: 10.1186/1471-2407-12-562 (PMC3530429; doi:10.1186/1471-2407-12-562)
Supplement: Additional file 2: — Figure S1. Metastasis free survival likeliehood statistics as described by Prat et al., (2012). To compare the amount of independent prognostic information provided by Ep-CAM (A) and AURKA (B) we estimated the likelihood ratio statistic in a model that already included AURKA (A) or Ep-CAM (B). The model shows that AURKA provides significant additional information over grading in the cohort of all patients, as well as in the ER+/HER2- subgroups (B). Vice versa, Ep-CAM provides additional information over AURKA only in the cohort of all patients. (PPT 182 kb) [file 1471-2407-12-562-S2.ppt]

## Slide 1
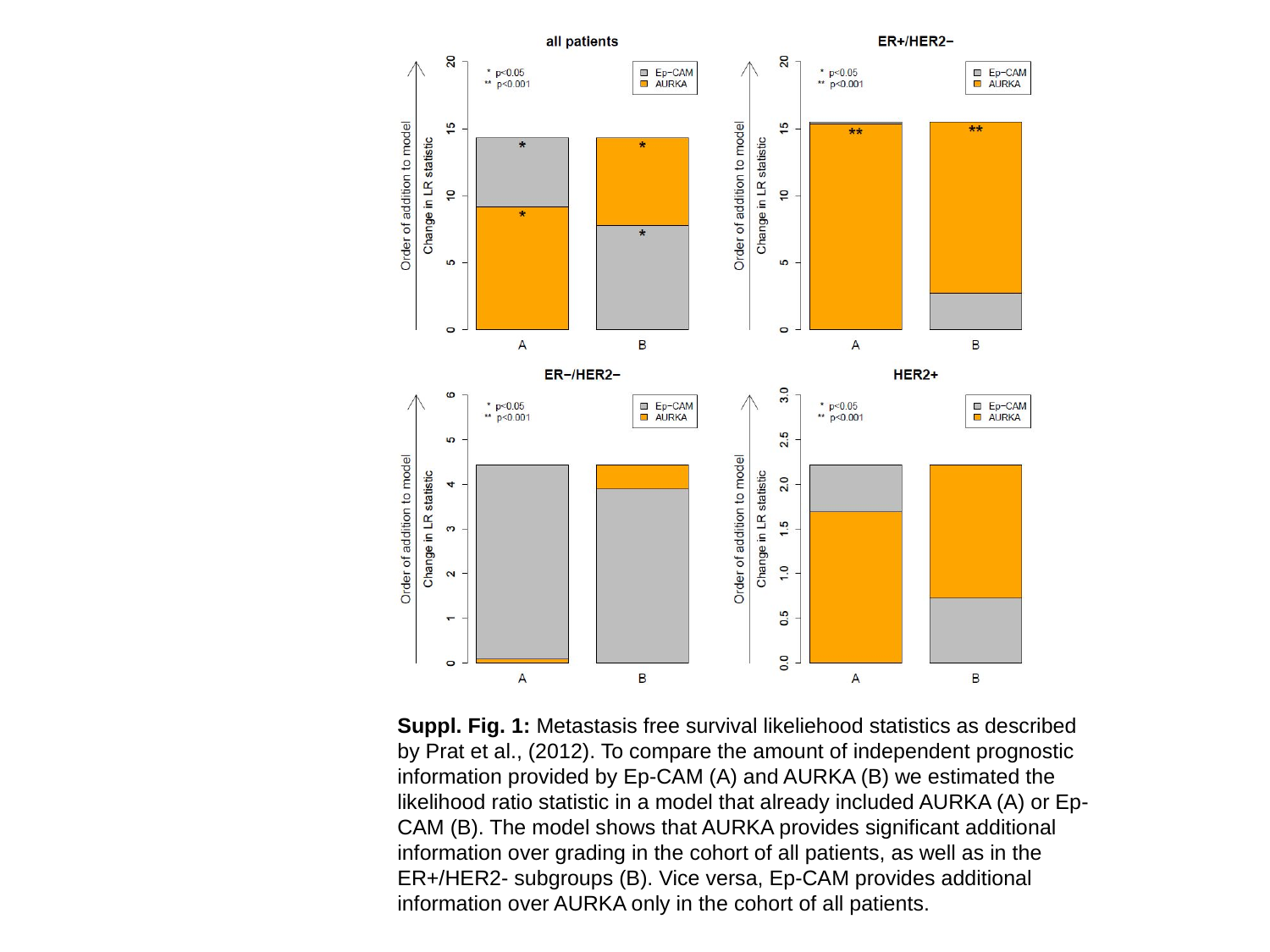

Suppl. Fig. 1: Metastasis free survival likeliehood statistics as described by Prat et al., (2012). To compare the amount of independent prognostic information provided by Ep-CAM (A) and AURKA (B) we estimated the likelihood ratio statistic in a model that already included AURKA (A) or Ep-CAM (B). The model shows that AURKA provides significant additional information over grading in the cohort of all patients, as well as in the ER+/HER2- subgroups (B). Vice versa, Ep-CAM provides additional information over AURKA only in the cohort of all patients.
